# Understanding the Effects of Music Care on the Lived Experience of Isolation and Loneliness in Long-Term Care: A Qualitative Study

**DOI:** 10.3390/healthcare10030457

**Published:** 2022-02-28

**Authors:** Sheetal Cheetu, Mara Medeiros, Lauren Winemaker, Maggie Li, Lee Bartel, Bev Foster, Chelsea Mackinnon

**Affiliations:** 1 Faculty of Health Sciences, McMaster University, Hamilton, ON L8S 4L8, Canada; cheetus@mcmaster.ca; 2 School of Communication Sciences and Disorders, Dalhousie University, Halifax, NS B3H 4H7, Canada; medeim1@mcmaster.ca; 3 Rehabilitation Sciences Institute, University of Toronto, Toronto, ON M5G 1V7, Canada; lauren.winemaker@mail.utoronto.ca; 4 Faculty of Medicine, University of Toronto, Toronto, ON M5S 1AB, Canada; maggieheyi.li@mail.utoronto.ca; 5 Faculty of Music, University of Toronto, Toronto, ON M5S 2C5, Canada; lbartel@chass.utoronto.ca; 6 Room 217 Foundation, Port Perry, ON L9L 1A5, Canada; bfoster@room217.ca

**Keywords:** music, long-term care, caregiving, non-pharmacological interventions, loneliness

## Abstract

This qualitative study aims to understand the lived experience of residents and other stakeholders during the implementation of a comprehensive music program in long-term care. It was conducted using a subset of 15 long-term care homes from the Room 217 Foundation Music Care Partners (MCP) “Grow” study in Ontario, Canada. The MCP program’s approach to music delivery uses therapeutic music practices such as “music care” to improve the care experience for caregivers and residents in long-term care homes. Thirty-two participants were interviewed, including staff, volunteers, and residents. Data were transcribed and analyzed using a modified grounded theory approach based on emergent themes. In total, seven themes arose from the data: limited resources, distinct experiences, life enrichment, dynamic relationships, program flexibility, potential continuity, and enhanced socialization. This study provides insight on barriers, enablers, and outcomes of the MCP program and on key considerations for implementing a novel interdisciplinary music program in a healthcare setting.

## 1. Introduction

Long-term care is an important sector of healthcare in Canada, servicing some of society’s most vulnerable individuals. Currently, Ontario long-term care homes (LTC) support over 115,000 individuals by providing a home environment, continuous nursing care, and primary medical care [1]. In the 2016 Census, seniors over the age of 65 made up 16.9% of the population [2]. As this population continues to grow, it is projected that 199,000 new beds will be needed to support individuals in need of long-term care in Canada by 2035 [3].

The majority of residents in long-term care require focused support for various challenges and impairments. The 2018 Canadian Institute for Health Information’s Continuing Care report found that 90% of residents in long-term care have some form of cognitive impairment, while 86% need extensive support in daily living activities [1]. Despite the need for round the clock care, lack of staffing is one of the major challenges LTCs across Canada must face in providing quality care to residents. In the 2018 Ontario Long-Term Care Association’s member survey, 80% of respondents stated that they had difficulty filling shifts in homes [1]. Staff in LTC homes are usually overworked, and since shifts are left unfilled, residents do not always receive the care that they need [1]. A recent study from Ontario, Canada, associated increased staffing hours and nursing assistance per resident, with a higher quality of resident care in a population where the majority of residents had cognitive impairments, difficulty with activities of daily living, and dementia-related disease [4]. Recently, the unprecedented COVID-19 pandemic has caused LTCs in Ontario to suffer greatly. Currently, four out of five COVID-19 deaths have occurred in long-term care [5]. One of the main concerns in the homes is the increase of staffing shortages. The government of Ontario has made changes to requisites for employment to help alleviate these shortages. However, redeployment and temporary staff could result in rapid disease transmission and dire consequences for long-term care populations if daily care needs are not being met [6]. The recent Canadian Armed Forces report on the state of long-term care homes in Ontario during the COVID-19 pandemic reported severe understaffing, as well as aggressiveness and poor communication amongst staff [7]. The current state of long-term care affects the wellbeing of residents in these homes. For residents with dementia, changes in their social environment, such as staff changes, are associated with negative outcomes, such as an increase in mortality, a decline in physical wellbeing, agitated behaviour, and anxiety [8].

Currently, antipsychotics are used in LTC homes for residents with symptoms of distress, a practice that may be exacerbated by staffing shortages [8]. In many cases, residents with aggressive symptoms due to other existing medical illnesses, pain, personal need, and environmental factors are also administered antipsychotics [8]. In a 2017 USA qualitative study, long-term caregiver interviewees believed that medication was the most effective intervention in reducing symptoms of distress with ease [9]. Despite the caregiver’s beliefs, the US Food and Drug Administration (FDA) has emphasized that antipsychotics are not approved for dementia-related psychosis treatment, with a risk of mortality in elderly patients with dementia-related psychosis 1.6–1.7 times greater than the placebo [10]. In Canada, LTC homes have started to look to non-pharmacological interventions for distressed behaviours. The Canadian Foundation for Healthcare Improvement (CFHI) has worked with 56 long-term care homes across Canada to reduce the use of antipsychotics [11]. In Ontario, the usage of antipsychotics to reduce distressed behaviours has decreased from 35% in 2011 to 20.4% in 2017 [12]. Despite this decrease, Ontario has the highest percentage (23%) of worsening depression symptoms in long-term care residents across Canada [12]. There is an urgent need for social interventions to aid in reducing distressed behaviours.

Alongside distressed behaviour, there is a high prevalence of loneliness in residents of long-term care homes. Although there is limited research on the prevalence of loneliness in Canadian LTC homes, a study conducted in Finland nursing homes found that 9% of residents suffered from loneliness “often” while 26% suffered from loneliness “occasionally” [13]. Interacting with others can especially present challenges for residents with cognitive impairments such as dementia. Difficulty in communication as a result of language decline in residents with dementia is a significant predictor of increased levels of loneliness and reduced engagement in social activities [14]. This could be an important contributing factor to the prevalence of loneliness in LTC homes around the world. A related construct is social isolation, which is a measurable construct of the number of interactions one has with others [15]. Social isolation in the elderly has been associated with a progressive decline in cognitive and physical abilities. The 2014 National Seniors Council—Report of Social Isolation in Seniors pointed to evidence that social isolation had effects on the health of seniors, including increased risk of mortality, higher levels of depression and suicide, and increased cognitive decline [16]. The report also found that there was an increased risk of social isolation in individuals with dementia. Addressing social isolation and loneliness in long-term care can positively influence health-related outcomes and quality of life for residents. Downstream effects of decreasing isolation and loneliness may include decreased symptoms of distress, a slowed rate of cognitive decline, and potentially decreased burden on caregivers. Additionally, addressing social isolation and loneliness may provide a valid alternative to the introduction of antipsychotic medications. 

Music is a non-pharmacological tool that can be used to elicit positive social outcomes in the LTC context [17]. “Music care” is defined as the intentional use of music by anyone to enhance the quality of care provided [18,19]. Associations exist between engagement and decreased odds of loneliness when examining receptive arts activities for older adults [20]. A 2018 systematic review found that the most common outcomes of LTC residents engaging with music care were increased socialization or communication (18%) and reduced depression (12%) [17]. Furthermore, changes in behaviour and mood in residents with dementia have also been associated with music interventions [21]. In adult day centers, researchers found that music interventions were associated with mood improvement and reduced agitation in individuals with dementia [22]. The study reported significant differences in engagement and joy post intervention [22]. Researchers considered music interventions an affordable tool that were able to improve the daily experience of individuals with dementia. In addition to social outcomes, the implementation of music interventions have also been associated with increased brain activation (cognitive outcomes) in individuals with dementia. A recent study from Utah used functional MRI to examine individuals with dementia following a personalized music-listening program [23]. Researchers found that preferred music listening showed activation in regions associated with music memory (supplementary motor area) alongside an increased functional connectivity in corticocortical and corticocerebellar networks [23]. Music interventions have also been associated with a reduction in the use of antipsychotic medication. A recent US nursing home study observed a significant increase in the percentage of residents that discontinued antipsychotic medication (17.6% to 20.1%) after the implementation of an individualized music listening program [24].

Typically, in LTC settings, therapeutic and purposeful music is delivered by externally sourced contractors such as paid “entertainers” or music therapists. The music care partners (MCP) program was initiated by the Room 217 Foundation, a Canadian health-arts organization, and whose development was funded by the Ontario Trillium Foundation (OTF). MCP was designed to flip the traditional model of music delivery within LTC by supporting an internal “site team” as they implement a purposeful music care initiative within their home. The MCP program recognizes that music can be used to combat a diverse set of “challenges” in LTC, including isolation and loneliness, in a cost-effective and person-centred way. The MCP program includes a two-day training for internal staff on the use of music in LTC, and expert guidance and support (i.e., consultation) with a music therapist or equivalent as the home work to implement intentional music practices to decrease the isolation and loneliness of residents.

The MCP offers a unique approach to music delivery in LTC because it recognizes that therapeutic music practices (i.e., “music care”) can be implemented alongside other LTC staff routines to improve the care experience for both the caregiver and the care receiver. This approach is aligned with Odell-Miller’s position that professional practitioners (such as music therapists) and other care providers (such as musicians, staff, family members) can together provide the most holistic (and therapeutic) musical experiences [25]. The MCP model was piloted by the Room 217 Foundation in a study funded by OTF in 2017. This study led to the conceptualization of the “integrated model of music care” (IMMC), the theoretical framework developed to understand the process by which music becomes integrated into pre-existing health systems. There is an important interplay between theory and practice. The current MCP study will develop the understanding of both MCP outcomes and its theoretical underpinnings [26]. The IMMC is built around the fact that music is one of the most diverse tools that can be applied in healthcare contexts. Music impacts all human domains (unlike pharmacological interventions which primarily impact biological and cognitive domains) and can therefore be applied in care practices that address a diverse set of goals and outcomes. The IMMC recognizes that music is not a prescriptive, “one-size-fits-all” approach and invites communities and caregivers to design purposeful music care initiatives to meet the specific needs of their setting.

Despite staffing challenges and shortages in LTC homes in Ontario, the implementation of the MCP program significantly decreased self-perceived isolation scores of participating residents [27]. In addition to understanding the resident outcomes associated with MCP, it is critical to gain an understanding of the lived experience of staff, volunteers, and residents who were part of the MCP initiative, especially given the systemic challenges (such as staff shortages, caregiver burnout, etc.). The need to qualitatively investigate enablers and barriers of music interventions has been identified [25,28]. As such, the purpose of the current investigation is to elucidate the positive and negative aspects of the MCP program to inform its future delivery by examining the impact of introducing the MCP into LTC settings, from the perspective of residents and caregivers.

## 2. Materials and Methods

### 2.1. Overview

The Room 217 Foundation initiated the MCP “Grow” study to implement the Partners program in 24 LTC homes in urban settings in Ontario, Canada. A participatory action research (PAR) approach was utilized to integrate music into the LTC setting in a customized way that was appropriate for the unique context of each participating home. This current qualitative study was conducted with a subset of 15 MCP “Grow” homes to understand the lived experience of residents and staff who were affected by this project. An exploratory approach was utilized within the qualitative component of MCP in order to understand the full scope of positive and negative effects of introducing the program in the LTC setting.

### 2.2. Study Setting

Residents, staff, volunteers, and family members from 15 LTC homes from 3 cities in Ontario, Canada, participated in this analysis. Eight homes were located in urban settings (in Toronto and Hamilton, ON, Canada), five homes were located in suburban settings (in Toronto, ON, Canada and Hamilton, ON, Canada), and two homes were located in a rural setting (in Cambridge, ON, Canada).

### 2.3. Design

Participatory action research recognizes the community in which research takes place as valuable collaborators within the research process [29]. PAR is built around the idea that research results are only meaningful if they make a difference to the individuals who implement the change, or those who are affected by it [29]. As such, each LTC community was invited to design their own “music care initiative” that would meet the specific needs of residents and staff within the community. Outcome data were collected pre- and post- implementation of the music care initiative to understand the impact of music care on residents’ lived experience of isolation and loneliness. These quantitative results are reported elsewhere [27].

Qualitative interviews were integrated into the PAR process at each LTC home, because feedback from the community of study is a critical component of data collection within PAR. Specifically, residents and staff members were invited to participate in an open-ended interview about their experience with the MCP program.

### 2.4. Music Care “Initiative” Intervention

The interviews were centred around the lived experience through the MCP program. As previously discussed, each participating LTC home implemented a two-month music care “initiative” (MCI) with the goal of decreasing isolation or loneliness in participating residents. With the support of a music care expert (such as a music therapist or equivalent), each LTC home set up an initiative that best fit the needs of their specific community. While a music care expert was available to assist in the design and troubleshooting phases of intervention development, each LTC home was responsible for implementation of their chosen intervention (independent of this external point person). An internal “site team” of staff, residents, volunteers, and community members was assembled to assist with this process at every home. For example, one LTC home created a music care initiative centered around individual music plans tailored to the needs of participating residents. Another home placed ideas for musical moments in a jar and had staff spontaneously pull the ideas out and execute them throughout the day. Various choir or singing group initiatives were also created, where residents would gather together to sing, play instruments, and converse with each other. Table 1 includes a description of all the home-specific interventions implemented by LTC homes participating in the study.

### 2.5. Participant Selection and Recruitment

All 24 LTC homes participating in the MCP “Grow” study were invited to participate in the qualitative interview process. Fifteen homes chose to participate, based on their individual capacity to engage in this qualitative research. The site team leaders (i.e., the staff member who was responsible for leading the MCP program at their LTC home) were provided with information about the qualitative interviews and asked to invite resident(s) and staff to participate in this aspect of the research process during the latter half of the two-month implementation phase. Interviews took place between weeks 4 and 8 of the initiative implementation period, or directly following the implementation period (i.e., weeks 8–10). Residents who participated in the interviews had to have been involved in at least a moderate way in the MCI. Similarly, participating staff were either site team members or intimately involved in the process of delivering music care at their LTC home. In some cases, site team leaders suggested interviewing a community volunteer or family member who was involved in the initiative. The research team trusted the judgement of the site team leader, in terms of which community members would be able to provide valuable insight into the lived experience of the project. In this way, the qualitative analysis relied on convenience sampling for recruitment methodology.

It is important to note that the residents represented in this sample have a higher cognitive capacity than the mean cognitive score of participating residents in the MCI. The current sample is biased towards higher cognitive capacity as these residents were chosen based on their cognitive capacity and ability to critically analyze their experience throughout the program.

### 2.6. Interviews

The three student researchers were responsible for conducting all the qualitative interviews throughout the two-year project. The goal was to interview at least one resident and one staff member involved in the Partners program/research at each LTC home. There were two interview protocols, one for residents and another for staff members. The protocol for staff interviews was to ask, “what are the barriers and enablers of music care implementation at the level of the organization, infrastructure, and individual/person-level interactions?” The protocol for resident interviews was to first ask, “how are you finding the music program?” The residents were then prompted to discuss their experience with the music program in more detail with the statement, “tell me a bit about your experience with music at your long-term care home.” If the cognitive capacity of the resident was high, the interviewer was provided the option of asking additional questions by following the protocol for staff interviews.

As such, the interviews were open-ended and flexible in terms of length and content. The goal of the interviews was to understand their lived experience in the Partners program.

### 2.7. Data Analysis

Qualitative data analysis was conducted using the software N-Vivo. Analysis followed a top-down approach using a modified grounded theory qualitative method [30]. The qualitative analysis team consisted of seven individuals who had varying amounts of exposure to the Music Care Partners program. It was critical to have team members with a variety of exposure levels to the Partners program to ensure that biases of the individuals intimately involved in the program delivery were recognized and checked during the analysis process. Specifically, the team consisted of the executive director of the Room 217 Foundation (the not-for-profit organization that developed MCP), the MCP project manager, three MCP program student research leads, and two additional undergraduate students who had minimal experience with the delivery of the MCP program.

Themes or core concepts arose from recorded and transcribed interviews conducted in long LTC homes. Student research leads selected three interviews that stood out to them as being “rich in content”, defined by the research team as interviews where interviewees consistently contextualized their responses with examples and critical reflection was present. These nine interviews were reviewed for overarching concepts and served as the initial observational data to find the preliminary code structure and themes by a student researcher with minimal experience with the delivery of the MCP program. Observations in the data were annotated as memos and later coded using identifying anchors, or codes. Coding followed an open approach, and data were directly analyzed for emerging themes. Memos varied in size and depth and were used to adjust, create, or combine initial themes. Initial themes remained broad and inclusive to ensure the inclusion of all noted observations. An initial theme list was explained and defined by the team member to identify the reasoning and interpretation of the various themes.

Each remaining data analyser coded between 5–10 interviews using the initial theme list as a framework. Analyzers coded under the initial themes and grouped similar codes into categories. The team came up with 39 coding categories, in addition to the original 10 themes. Following the coding, the initial themes list was adjusted by redefining themes to better include all found categories.

Seven overarching themes were finalized by the entire team, including all categories as subthemes. To consolidate these themes, specific codes were revisited to ensure the new descriptions of the themes aligned with the observed data. The themes and sub-themes were re-evaluated based on the codes they contained. Subthemes “creativity” and “organization” were removed as they did not reflect the program specifically. Certain subthemes were also added, such as “involving more residents” and “creating more initiatives”, to group specific codes under a broad theme.

## 3. Results

### 3.1. Participants

Thirty-two individuals were interviewed across 15 different LTC homes (Table 2). There were 27 interviews conducted as three interviews were conducted in a group of two and one interview was conducted in a group of three. Nineteen (59%) interviewees were female and 13 (41%) were male. Eleven of the interviewees were residents, and the remainder were staff members or volunteers of various titles. The staff members interviewed were interdisciplinary and were involved in their respective music care initiatives to varying degrees (Table 3).

### 3.2. Themes

Seven overarching themes emerged from this qualitative analysis. Table 4 reports the number of codes attributable to each theme, as well as the number of interviews that included at least one code related to the overarching theme.

Theme 1: Limited Resources

“Resources can be an issue….”

The first overarching theme is limited resources. Interviewees consistently stated that a lack of resources was one of the greatest barriers to the MCP program as it prevented the optimization of music care delivery in LTC homes. Subthemes emerged based on the types of limitations: funding, buy-in, technology, and physical space. Staff interviewees found that limited funding affected the quality and quantity of resources that were needed to supplement the music care initiatives, such as musical instruments:

“So we realized we needed to order a second set of bells. They were ordered eight days after the first set, and they have not arrived.”

In some cases, funding did not account for the number of participating residents. A lack of buy-in occurs when key players do not fully embrace the MCP program, which ultimately decreases the amount or quality of music care delivered to residents. Some interviewees described a lack of buy-in from staff members, as staff were resistant towards the initiative, and had little incentive to participate:

“Are we gonna pay them to stay an extra hour every day or something like that? So we ended up not getting any of the staff.”

In this instance the interviewee discussed the need for incentives to have staff participate. Staff had various duties to perform inside the home, and in some cases felt uninclined to facilitate initiatives. For example:

“[Getting the nurses on board] was a little bit more difficult to achieve. They, at first, look at it as a recreation job. Like you guys are the ones who program so you guys do it.”

This interviewee noted that staff perspectives and expectations of the program played a role in how willing they were to participate and run initiatives.

Technology also played a significant role as a resource for MCIs. The lack of quality of music players and up to date technology detracted from the MCI experience by affecting the delivery of MCIs. Interviewees found that technological issues consumed program time and affected how well residents could participate:

“[The music system] does have some problems. Sometimes it charges and takes like ten to fifteen minutes to get it going. So it’s a little bit different.”

Specifically, this interviewee describes the technological problems that occurred, which resulted in lost time during the initiative. Lastly, physical space played a role in how well the MCIs could be implemented:

“Ideally like there should be a different space in which we can practice but there is just limited spacing here.”

In some cases, long-term care homes felt limited in the space they had to run MCIs and voiced that a larger space would better accompany the program.

Theme 2: Distinct Experiences

This theme is based on the idea that individuals experience the MCP program and are affected by music differently. In other words, the experience of music care is highly specific to the individual. Sub-themes include individual barriers and relationship to music. ‘Individual barriers’ addresses how the residents’ experience is impacted by factors such as cognitive, and physical capacities, as well as day-to-day fluctuations. Certain initiatives were impacted by cognitive and physical barriers more than others. For example, one interviewee noticed that residents with cognitive and physical impairments had difficulties participating in the home’s bell choir.

“Yeah, because some people are really struggling, just maybe, either cognitively or physically, in ringing the bell properly.”

Another interviewee noticed that higher functioning residents were able to verbally participate in music care, whereas participation from lower functioning residents was more subtle.

“Well, it’s verbal, and it’s different. But it doesn’t mean that like it… That’s why I think reports and that’s why my notes I think are really important. Yeah. Because if I can start noticing somebody’s foot tap, right. And I can see somebody’s hand starting to go to a beat, right? Like things like that. But then I have to see that and write that down in order for that… because that is a piece of information important.”

Certain music care initiatives were tailored to higher functioning residents due to the greater response received from these individuals.

“And the third floor has a higher level of functioning. Right. So I think this kind of a program has kind of automatically morphed to this floor, simply because of level of ability. You’re going to get more response.”

Responses to music care not only varied amongst individuals, but from day to day within an individual as well.

“Day to day, it’s different in long-term care, you can have moment to moment differences. I had a gentleman who was really animated and contributed wonderfully last week. He had a bad night. Didn’t have a good morning, halfway through, he had to use the washroom. He just wasn’t himself and fell asleep a lot. That’s just the nature of long-term care, right? And then I had a lady who this morning felt very sad, didn’t want to come out of her bed, and I can’t force someone to come.”

The relationship to music subtheme addresses the residents’ individual music preferences, level of musicianship, and preferred approach to engage with music. Some residents use music as a social point to engage in conversations, others like to play along, and some just want to listen. The staff help make this individual experience possible by reaching out to residents and trying to figure out the best approach to get the individuals engaged. One interviewee commented on how using specific music that was meaningful to one of the residents helped facilitate a reduction in behaviours.

“There’s a particular resident on one of the units, every time there is a behaviour, I use the same music. So it’s actually one of the Christmas ones, the holiday season. I guess she kind of connects with and is familiar with the Christmas songs.”

A number of interviewees commented that reminiscing was a common way residents would engage with music.

“Because they’ve said it themselves that this brought back memories… thinking either about the war or when they were younger.”

“He brought in a picture one time of his family band when he was younger.”

“Yes, we chatted about what it was, why it was their favourite song and what we liked about it. Some people would share some things that happened to them.”

Theme 3: Life Enrichment

Life enrichment addresses the personal impact and changes of music care on participating residents. The two subthemes emerged based on the types of changes interviewees observed: mood changes and behavioural changes.

In many interviews, mood changes, such as levels of enthusiasm and excitement among participating residents were observed by interviewees:

“[Prior to the start of the program] I don’t have to be playing music I don’t have to have anything in my hands but they’re excited.”

Similarly, residents also identified music’s capacity to change mood:

“We sing songs that we like at the table (me and my tablemate). It gets our mind off of things.”

Interviewees observed that residents were engaged and excited to participate in the programs. Alongside excitement, interviewees noted changes in irritability and increased calmness as a result of the program:

“Another resident, she’s kind of on the angry side a lot of the time and I’ve noticed that she will listen to Elvis or Bobby Darin on the iPod and sing and dance.”

In this case the staff interviewee noticed changes in a resident’s mood when listening to music. Although she was otherwise irritable, she enjoyed listening to music and stayed calmer.

Alongside mood changes, interviewees noted changes in behaviour such as increased sociability, increased activity, and increased eating during mealtimes:

“I also include music in the background so the music to release to decrease muscle tension and to help it to stay calm to motivate them to do the exercise.”

Staff interviewees mentioned the successful use of music to motivate residents to participate in physical activities.

“I’ve noticed a lot of changes in the residents over time. I think they’re more relaxed [during] meals.”

Interviewees observed changes in behaviour patterns around the homes including more consistent eating patterns in residents.

Theme 4: Dynamic Relationships

This theme encompasses the impacts of music care on relationships and the impact *of* relationships on music care delivery. There are many types of relationships in the LTC setting, including resident-staff, resident-resident, resident-family, staff-staff, among others. This theme addresses the way that music care and the MCP program impacts relationships, as well as how relationships impact the delivery of music care. Subthemes include quality of relationships, compassionate caregivers and communication.

Throughout the interviews, it can be shown that the residents’ relationships with the staff have grown stronger over time. Music was used as a tool to enhance the quality of the relationships between residents and staff.

“You know myself, my supervisor and the rest of the people participating in it… You just we all love music. We express the same reaction to music; we just get engaged right away. Music is bonding.”

“If the staff is included that will help the residents to feel more connected to the staff as well as other residents.”

The compassionate caregiver sub theme is exemplified in the following quote. As outlined by the theme definition, music has shaped the lives of residents in different ways. When a caregiver takes the time to understand a resident’s connection to music, it allows music to be used in a purposeful and meaningful way. Caregivers taking the time to get to know the nuances of each resident’s experience and personality showcases their compassionate nature.

“For example, I have one gentleman who loves music, and every time I see him, our connection with each other is talking about music. Which is awesome. And for him, music can be a daily thing. But someone who, music wasn’t part of their lives, or was just on Sunday at church, which is a reality for this age group and this demographic as well. [Music] was a good thing and a joyful thing and something that brought happiness and a comfort level for sure. But they didn’t necessarily go to dances and [music] wasn’t taught. And then I have a lady who danced ballroom dance since she was three years old and loves all kinds of music and music is naturally a part of her life. Not because I brought it or because I brought her to music appreciation, simply because music always was for her.”

In comparison, when strength in relationships between staff groups was lacking, there was a significant impact on the music care initiative. Interviewees felt that communication was an essential component of staff–staff relationships, and a lack thereof was a barrier to the implementation of successful music care delivery. Interviewees explained that improved communication could help eliminate confusion about tasks (i.e., two staff members performing the same task) and help the entire home have a cohesive understanding of music care.

“Communication becomes a barrier from time to time, and I think at this home it was.”

“Um, I think the communication piece, maybe we fell short on that a little bit, is trying to help the staff who weren’t trained, to at least come in for a mini thing and see what it’s about. I don’t know whether that was done. I wasn’t called into it. I think that would have been a beneficial piece.”

Theme 5: Program Flexibility

Program flexibility addresses how the structural variations within the different music care initiatives allow for a better adaptation of the program in varying care contexts. Different MCIs had different levels of flexibility and/or adaptability. The essence of music care is flexibility, and it is designed to be adapted for specific care contexts. Subthemes of adaptability, expectations, and benefits of MCT showcase the different levels of flexibility that participating homes achieved. Adaptability allowed for MCIs to follow a person-centred approach. Programs were made to better accommodate and engage residents with varying needs.

“Our [program] is very approachable. We don’t put a restriction on them, we tell them that it’s fun. So it’s not necessarily all regimented we just let loose and we just play.”

Specifically, this interviewee discussed the importance of approachability in their program. This approachability attracted residents and allowed them to engage in music without restrictions. However, since programs were flexible in nature, in some cases, expectations played a role in how facilitators of the program engaged the residents.

“I feel like I didn’t really get really good learning and direction on what was expected. From my understanding, I sort of turned it into this because this was the only way that I could figure out how I could do music appreciation in a group.”

In this case, the interviewee felt overwhelmed with the open nature of the program, which resulted in a specific program structure. Despite this, music care training was able to reduce overwhelming expectations and foster creativity and flexibility within the program.

“[The music care trainer] was absolutely wonderful and I loved how passionate she is about music, and how she opened my eyes to I just loved some of her ideas like, like using a familiar song changing the words to try to motivate somebody I thought was just divine and, and fun…Some of the tools that were taught to us were fabulous.”

Interviewees with training in music care utilized tools to add to the adaptability of the program and felt more invested and engaged in the initiative themselves. Tools described were specifically to help motivate residents to engage in the initiatives, rather than tools specific to the structure of the program.

Theme 6: Potential Continuity

“I think that was the most exciting thing is [that] you can probably do more of this even after the project is over.”

Music care initiatives have the potential to provide long-term benefits through continuation and integration in long-term care homes. Integration of music care can be seen through the “ripple effect” phenomenon which occurs when music care is expanded and spreads beyond the scope of this project. The “ripple effect” can manifest in three ways: involving more residents, creating more initiatives, impacting more challenges. Some MCIs attracted more residents than anticipated, as the initiative grew more popular throughout the home:

“One said she only liked classical music, but she was coming out and she was willing to listen to other music.”

This interviewee saw that otherwise uninterested residents wanted to participate and engage in the music initiatives although they had different tastes. Music care could be seen outside of the original initiative, and staff wanted to see the long-term implementation of music care in the long-term care home:

“Above and beyond this initiative we’ve tried to just include a lot more music around the home in our common areas.”

A staff interviewee discussed efforts in their home to include music care in a variety of ways, including integrating music in more spaces. Music care was used to target not only loneliness and isolation but also other challenges such as sleeping and changes in attitude and behaviour:

“Sometimes in 35 to 40 min, the resident can go from having behaviours to sleeping.”

In this case, the interviewee observed changes in a resident’s behaviour as a result of music care. The ripple effect is an indicator of the potential continuity of music care in homes and was seen often in the data.

One interviewee explained that although they faced interruptions due to the COVID-19 pandemic, they are eager to continue music care when it is feasible to do so.

“We’re hoping to continue it and we will, eventually, but we can’t get all the people together at the same time to do much with it, you know?”

Theme 7: Enhanced Socialization

“[A resident who is otherwise] secluded, and doesn’t come out, he’s coming to listen, he’s going to the social hour on Thursday night…”

An important effect recognized by multiple interviewees is enhanced socialization, and this theme encompasses how participants connect with each other socially as a result of the music care initiative. This was different than prior to the music care initiative, where individuals were lonely or stayed isolated. A staff interviewee noted that residents who would otherwise describe themselves as isolated have opened up as a result of the initiative:

“He kind of said that he’s a little bit of a loner…it’s been great that music has really been a way to open the door with him.”

MCIs create a space that fosters a group dynamic where residents feel connected:

“I get hyper at one minute and then it makes me laugh and joke with others.”

This quotation showcases the strength of music on socialization between residents.

Interviewees noted the group helped support and encourage individual participation in the program and enhance socialization.

“It’s making a difference. The residents who are coming out even if they’re not coming out regularly I think it’s getting some of them to open up more talk share more in a group.”

In this instance, interviewees observed residents feeling more comfortable sharing and engaging as a result of the group setting. After the implementation of the initiative, residents engaged in more group activities, and more conversation with others.

One resident explained that while he enjoyed the music itself, one of the greatest parts of the program was how music facilitated socialization and meaningful interaction. Another resident explained one of her favourite parts of the program was enjoying it with her tablemate.

“Music is good for us, and it increases the social aspect.”

Another resident explained one of her favourite parts of the program was enjoying it with her tablemate.

“[My table mate and I] sing songs that we like at the table. It gets our minds off things.”

## 4. Discussion

The MCP program was designed to decrease the experience of isolation and loneliness for LTC residents through music. Since the MCP program is a novel approach in LTC homes, it is critical to examine the lived experience of residents, staff, family members, and volunteers to understand the full scope of the impacts of the program. Understanding factors that played a role in program delivery will inform program modifications in an evidence-based way. This analysis will help to optimize the delivery of music care in the MCP program and the greater LTC community. Additionally, this analysis can provide insight into key considerations and factors when implementing a novel interdisciplinary music program in a healthcare setting.

Qualitative analysis was conducted using interviews to capture the lived experience of stakeholders within the program including staff and residents. Seven themes emerged as a result of this analysis: limited resources, distinct experiences, life enrichment, dynamic relationships, program flexibility, potential continuity, and enhanced socialization. Interestingly, the seven themes can each be categorized as outcomes, enablers, or barriers. A program outcome is a theme that emerges as a result of the initiative. This helps to understand the impact of the initiative on the long-term care home population. An enabler is a theme that addresses the process of the initiative and ultimately enhances the delivery of music care. A barrier is a theme that prevents the process or subsequent outcomes from occurring.

Limited resources were consistently a barrier to music care delivery at participating in LTC homes. While different types of resource limitations occurred in different LTC communities (i.e., funding, buy-in, technology and physical space), many interviewees expressed at least one type of resource limitation.

Distinct experiences was an outcome as it describes a result of the implementation of the initiative. In all interviews, the impact of music care on residents depended on subthemes: individual barriers and their relationship to music. Based on these subthemes, music care had a unique impact on residents. Residents experience the personalized impact of music care on residents.

Similarly, life enrichment was an outcome of the music care initiative. Staff and resident interviewees consistently observed changes in residents’ moods and behaviours as a result of the initiative. Staff interviewees also noted that this observed outcome motivated residents to exercise and regulate eating patterns.

The reciprocal nature of impactful relationships makes it both an enabler and an outcome. Relationships play a role in how well the initiative is received and implemented in the home. Staff interviewees in many homes noted that relationships between staff and residents created a supportive environment in which the initiative could thrive. Alongside this, the development and strengthening of relationships also occurred as a result of the program. Staff interviewees described their personal experiences in creating new connections with residents through music.

Program flexibility was an enabler to the initiative as it allowed for the program to supplement the distinct needs and experiences of the residents. Interviewees described varied approaches to music care, and only in very limited instances described their program structure as rigid and definite. Most interviewees acknowledged the lack of restrictiveness of the program structure, to ensure the inclusivity of all residents.

Potential continuity was an outcome of the MCP program. Most interviewees discussed the impact of music care on residents and the benefits of long-term implementation. Interviewees who did not feel that music care could be implemented long-term attributed limited resources as a barrier to continuity, not the impact of music on the residents. Furthermore, the ripple effect, an indicator of continuity, was observed in many interviews.

Enhanced socialization is one of the most common observed outcomes of the program. Interviewees noted that prior to the initiative, many residents stayed isolated and were not engaged with other residents. Music care acted as a catalyst for social connections. Interviewees noted increased resident-resident interactions, increased resident-group interactions, and even increased resident-staff interactions after the implementation of the initiative. The prevalence of enhanced socialization is in line with the quantitative findings that the implementation of the MCP program significantly decreased self-perceived isolation scores in participating residents.

It is important to recognize that two overarching themes that emerged from this analysis are directly related. Enhanced Socialization is an outcome and occurs when residents are more social with other LTC home community members (including residents, family members, staff, volunteers). The subthemes of dynamic relationships “quality of relationships” and “communication” can be enablers, barriers, and outcomes of the MCP. While some codes contributed to both of these themes, enhanced socialization is its own theme because of the high prevalence of discussion of socialization as an outcome of the MCP.

Behavioural changes is a subtheme of “life enrichment” and relates to resident outcomes and responses that occurred as a result of the MCP program implementation. The subtheme ripple effect (under the overarching theme “potential continuity”) can manifest in three different ways, one of which is through behavioural changes occurring for residents who were not formally recruited or participating in MCP. This indicates a ripple effect because the impacts of the MCP are extending beyond the original scope of the program. As such, the subtheme “behavioural changes’’ refers to changes occurring within the subset of officially participating residents, and “ripple effect” behavioural changes occur when residents outside of the scope of the original project are impacted.

LTC communities and researchers can utilize the outcomes from this analysis when implementing the music care approach in healthcare settings. Prior to the implementation of music care, communities can assess the resources available, including staff, technology, and monetary. In particular, the amount of buy-in that a LTC home is able to leverage is a critical “resource” that will impact the delivery of music care. Although limited resources were consistently barriers in this analysis, LTC homes have an opportunity to use resources as an enabler for music care delivery in the future. Music programs do not have to be expensive, and the flexibility within the music care approach invites communities to work with what they have available to create a meaningful music program. Finally, the nature and strength of relationships within the LTC home can be examined prior to music care implementation, as these will impact the delivery of music care.

As LTC communities work to design their music care “initiatives” or programs, it is important to examine the amount of flexibility that will be possible within the initiative’s design. Recognizing that each resident will have a unique experience may also help during the design process. Finally, based on the experiences of previous LTC homes, future delivery of music care could use the outcomes of life enrichment, dynamic relationships, enhanced socialization, and continuation of the program as benchmarks for success.

This qualitative evaluation contributes valuable information to the “integrated model of music care”, which is a theoretical framework explaining the ways that MCP can be integrated into healthcare settings. Recognizing that music care is being introduced into systems and communities that have pre-existing structures, a critical component of this framework includes recognizing the lived experience of individuals within the community who will implement or experience music care. In particular, this investigation produced a set of enablers, barriers, and outcomes of the MCP delivery that will be invaluable tools predicting the success of music care implementation in future care communities.

## 5. Conclusions

Music may enrich quality of life for residents in LTC through the strengthening of relationships and social inclusion. In this way, music can change the culture and atmosphere within a LTC home for staff, family, and residents. Music can also be personalized, acknowledging the preferences of an individual, which may be influenced by geography and other factors. Music is a resource that is able to be compassionately used by all caregivers, no matter their formal musical background, day and night, in dining rooms, in hygienic care tasks, in palliative and end-of-life care, for transitions, through outbreaks and pandemics. When implemented with purpose and training, music can make a noticeable shift in attitudes, engagement, and behaviours. This study has shown that to truly sustain music care delivery and integrate it into daily life in LTC, staff need to be given permission and become equipped to look to music as a viable solution to systemic and very personal challenges like social isolation and loneliness.

Music needs to become a standard of care in every long-term care home in Canada. Funding for music must become available through government funded envelopes. There needs to be a full-time music therapist or equivalent as on-site expert that can provide leadership and ongoing training to staff. Resources like handbells, drums, sound systems, and other music care specific tools should not have to be fundraised or personally donated by staff, but rather provided and valued as important social necessities for communities of care looking for pharmacological alternatives that are cost effective and easy to use. Music is a human phenomenon, and there is no place at the present time that can use it with such profound effect as long-term care homes in Canada.

## Figures and Tables

**Table 1:** Intervention descriptions.

Home-Specific Intervention Description	Music Delivery	Frequency of Intervention
Participating residents play percussive instruments (such as djembes, shakers, drums) along to recorded music. Located in the hallway to invite others to join.	Live & recorded	10–20 min, 2–3 x/wk
Residents gather in a common space to listen to culturally or personally important music. Discussions about why the music is important to each participant follows the music listening.	Recorded with video (YouTube)	45 min, 1 x/wk
Spontaneous musical conversations, musicking, chimes, and sing-alongs facilitated by staff and volunteers.	Live & recorded	5 min, 3–5 x/wk
Music care plans were created for participating residents and integrated into the care planning system for use during 1:1 visits with recreation staff.	Live & recorded	10–20 min, 1–3 x/wk
A bell choir was created with 16 participating residents, and facilitated by a community volunteer. A coffee social followed each practice to enhance socialization between residents.	Live	60 min, 1–2 x/wk
Person-specific 1:1 visit plans that incorporated music were developed and implemented for at-risk isolated residents. Group music events building upon the individual sessions were offered.	Live & recorded	10–20 min, 2–4 x/wk
Every resident in the building (>275) contributed songs to a curated home-wide playlist to be used during mealtimes to create positive experiences and interactions in the dining rooms.	Recorded	20 min, 7–14 x/wk
Individual music care plans were created for at-risk residents. The care plans were implemented by members of the recreation staff team and include creating playlists, a musical knitting group, etc.	Live & recorded	10 min, 2 x/wk
A resident choir was created to increase resident:resident socialization and to create a unique community experience.	Live	30 min, 2 x/wk
Isolated residents were visited by recreation staff members to engage through music. Over time, staff and residents formed relationships and multiple residents would engage in each staff-led visit.	Live & recorded	15 min, 1–3 x/wk
Ideas for musical moments were placed in a jar and staff were invited to spontaneously pull the ideas out and execute them throughout the day.	Live & recorded	5 min, 3–10 x/wk
The Pathways Singing Program (a sing-along DVD set with associated recreation programs) was implemented in one area of the home.	Recorded	20 min, 2 x/wk
Musical activities were designed for residents who otherwise struggle to participate in daily life at the LTC home. Music interactions were focused on maximizing accessibility and inclusivity for participants.	Live & recorded	10 min, 2–3 x/wk
The “Singing Social” involves karaoke-style sing-alongs for residents and staff in a common area of the home. Participants may sing in a group or as individuals; they may accompany themselves, sing acapella or to a recording.	Live & recorded	45 min, 1 x/wk
Staff were taught three strategies to use in the morning, which has been identified as a difficult time at this LTC home: a wake-up song (written by the team), a familiar song, and musical activities.	Live	10 min, 2–7 x/wk

**Table 2:** Description of interviewees. Note that “Group of 2” refers to the percent of interviewees that interviewed in a group setting (i.e., two interviewees and one interviewer). Similarly, “Group of 3” indicates that three interviewees were present.

Participant Category	Percentage of Interviewees
Female	59%
Male	41%
Residents	34%
Staff members or volunteers	66%
Groups of 2	19%
Groups of 3	9%

**Table 3:** Positions of staff interviewees.

Staff Position	Frequency
Activation Facilitator	1
Programs Managers	2
Spiritual Care	1
Therapeutic Recreationist	1
Student Volunteer	2
Life Enrichment Manager	1
Life Enrichment Team	2
Program Therapist	2
Recreation Therapist	2
Dietary Team	1
Registered Practical Nurse	1
Physiotherapist	1
Activation Manager	1
Activities Team	1
Director of Programs	1

**Table 4:** Theme names, number of contributing codes, number of interview contributions.

Theme	# of Codes	# of Interviews
Limited Resources	35	17
Distinct Experiences	96	14
Life Enrichment	82	19
Dynamic Relationships	56	10
Program Flexibility	55	15
Potential Continuity	29	7
Enhanced Socialization	28	15

## Data Availability

The data presented in this study are available on request from the corresponding author. The data are not publicly available to protect the privacy of qualitative research participants.
